# Heterozygous frameshift *KMT2A* variant in a patient with Wiedemann–Steiner syndrome

**DOI:** 10.1038/s41439-026-00338-2

**Published:** 2026-03-17

**Authors:** Sawako Hirai, Hiroshi Mitsubuchi, Shirou Matsumoto

**Affiliations:** 1 Sophia Ladies Clinic Suidouchou, Kumamoto, Japan; 2 Fukuda Hospital, Kumamoto, Japan; 3 Department of Neonatology, Kumamoto University Hospital, Kumamoto, Japan

**Keywords:** Autism spectrum disorders, Disease genetics, Development

## Abstract

Here we report a case of a Japanese girl with Wiedemann–Steiner syndrome carrying a novel heterozygous frameshift variant of *KMT2A* (NM_001197104.2:c.10123del, p.Thr3375ProfsTer7). Her clinical features included severe pre- and postnatal growth failure, global developmental delay, hypertrichosis and complete agenesis of the corpus callosum. The identified variant truncates the protein, abolishes the C-terminal SET domain required for histone methyltransferase activity, and is predicted to trigger nonsense-mediated mRNA decay, resulting in *KMT2A* haploinsufficiency—the primary pathogenic mechanism of Wiedemann–Steiner syndrome. This report documents a previously unreported loss-of-function variant in *KMT2A* with detailed molecular interpretation and phenotypic characterization, contributing to refinement of the mutational spectrum associated with Wiedemann–Steiner syndrome.

Wiedemann–Steiner syndrome (WSS, OMIM #605130) is an autosomal-dominant neurodevelopmental disorder that was first described by Wiedemann et al. in 1989 and further characterized by Steiner and Marques in 2000^1,2^. De novo heterozygous pathogenic variants of the *KMT2A* gene (previously known as *MLL*) were first identified in 2012 as the underlying genetic cause through whole-exome sequencing^3^. WSS is characterized by a variable clinical spectrum, with the core features including developmental delay or intellectual disability, pre- and postnatal growth retardation, hypertrichosis, a distinctive craniofacial phenotype and specific neurodevelopmental profiles such as autism spectrum disorder with rigid behaviors^4,5^. The characteristic facial features often include thick eyebrows with a lateral flare, vertically narrow and down-slanting palpebral fissures, a wide nasal bridge with a broad or bulbous tip, and long eyelashes^6^. Hypertrichosis cubiti (‘hairy elbows’) was once considered a pathognomonic sign of WSS; however, large-scale genomic studies have revealed that this sign is present in only approximately 60% of individuals in whom the diagnosis of WSS was molecularly confirmed, highlighting the clinical variability of the syndrome^6,7^. The majority of reported pathogenic variants of *KMT2A* are loss-of-function (LoF)—encompassing nonsense, frameshift and splice-site alterations—which promotes the haploinsufficiency of the KMT2A protein as the primary disease mechanism^6,7^.

We describe a case of a Japanese girl with a constellation of features highly suggestive of WSS in whom a novel heterozygous frameshift variant in *KMT2A* was identified. The proband is the third child of healthy, nonconsanguineous parents with no family history of congenital anomalies or neurodevelopmental disorders. The prenatal course was notable for bilateral ventriculomegaly and suspected agenesis of the corpus callosum, detected via ultrasonography at 34 weeks of gestation. She was born at 38 + 1 weeks, weighing 2238 g (−2.1 standard deviations). She was 46.5 cm long (approximately the third percentile) and had a head circumference of 32.0 cm (slightly small for age). This finding is consistent with those of reports showing that approximately 45% of the patients with WSS exhibit prenatal growth retardation^6,8^. Although her postnatal oral intake was adequate, and complementary feeding was uneventfully introduced, she exhibited a persistent failure to thrive. Her growth consistently tracked below −3 standard deviations compared with the standard growth curves for Japanese girls (Fig. 1a). At 3 years and 7 months of age, she was 85.0 cm tall and weighed 9.55 kg. This severe postnatal growth retardation aligns with findings from large cohorts, where almost 68% of individuals experienced failure to thrive and 58% were short in stature^6^. The serum IGF-1 level of the patient when she was 1 year old was below the reference range, but the concomitant low prealbumin levels suggested nutritional insufficiency rather than isolated growth hormone deficiency. Nutritional counseling was initiated, and catch-up growth was monitored.

**Fig. 1:**
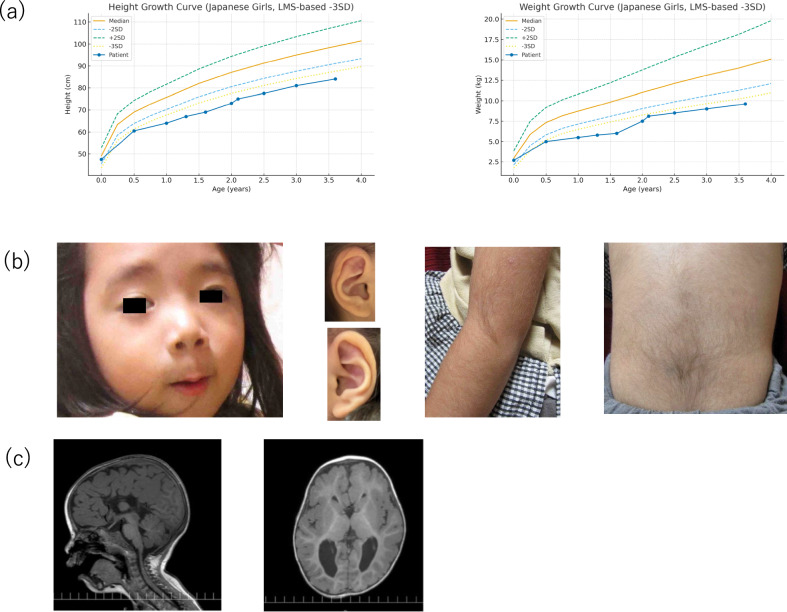
Clinical and radiographic features. **a** Growth parameters plotted on standard Japanese growth charts. The patient showed severe growth retardation in both height and weight. **b** Characteristic facial and physical features. Left: facial photograph showing distinctive features such as downturned eyebrows and right-sided ptosis. Middle: close-up views of the right (top) and left (bottom) ears, clearly showing the auricular structure. Right: hypertrichosis was evident on the elbow and back. **c** Brain MRI (sagittal T1 and axial T1) demonstrating complete agenesis of the corpus callosum with mild ventriculomegaly.

The patient experienced global neurodevelopmental delay. Major motor milestones were delayed, but within the spectrum reported for WSS. She was independently walking at 17 months, which is earlier than the median walking age of 20 months described in reported cohorts^6,8^. Language development was more affected, with single words spoken at 2 years but no two-word phrases spoken at 2 years and 7 months. This pattern is consistent with those described in previous reports showing that nearly all individuals with WSS (97%) exhibit some degree of developmental delay or intellectual disability^6,8^. Formal developmental testing at 2 years and 7 months using the Kyoto Scale of Psychological Development confirmed global delay, with a developmental quotient of 48. Physical examination revealed several facial features characteristic of WSS, including arched eyebrows, down-slanting palpebral fissures, right-sided ptosis and micrognathia (Fig. 1b). Brain magnetic resonance imaging (MRI) performed at 1 year of age confirmed complete agenesis of the corpus callosum with associated colpocephaly, a structural brain abnormality reported in a large proportion of patients with WSS^5,6^ (Fig. 1c). The clinical suspicion of WSS strengthened after the age of 2 years when hypertrichosis of the back and elbows became evident, a feature observed in 67% and 57% of patients, respectively^6^. In addition, the patient exhibited reduced sweating. Although this observation is based on a single case, it may warrant attention in future studies to determine whether sweat gland dysfunction represents a rare or underrecognized feature of WSS.

Exome sequencing identified a heterozygous single-nucleotide deletion in *KMT2A* (NM_001197104.2:c.10123del), resulting in p.Thr3375ProfsTer7 and truncation of the C-terminal SET domain (Fig. 2b). Although parental genetic testing was not performed due to clinical limitations, the parents are phenotypically healthy, suggesting that the variant probably arose de novo (Fig. 2a). This variant was absent from population databases (for example, gnomAD) as well as clinical databases (ClinVar and the Human Gene Mutation Database), supporting its novelty.

**Fig. 2:**
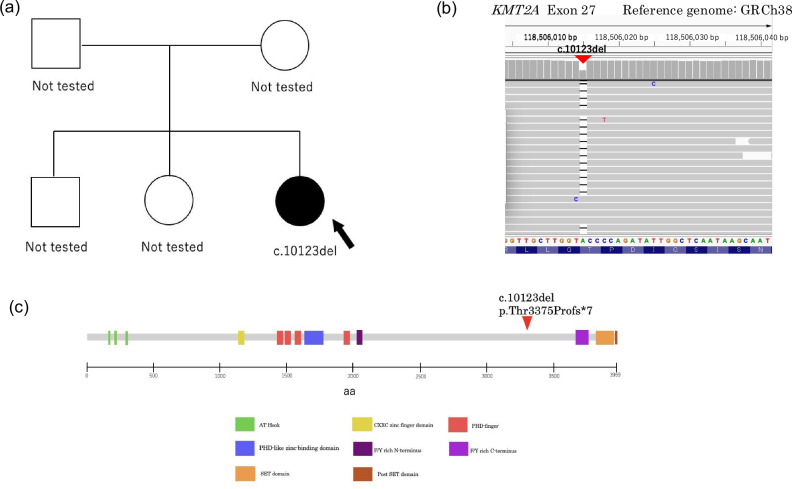
Genetic analysis of the *KMT2A* variant. **a** Pedigree of the family. The proband (arrow) is affected with the heterozygous frameshift variant. The parents are phenotypically healthy. Genetic testing for the parents was not performed (not tested). **b** Integrative Genomics Viewer (IGV) snapshot of the exome sequencing data from the proband. The image displays the heterozygous single-nucleotide deletion (c.10123del) in exon 27 of *KMT2A*, indicated by the red arrowhead and the gap in reads coverage compared with the reference genome (GRCh38). **c** Schematic structure of the KMT2A protein domains showing the position of the c.10123del variant (red arrowhead). The mutation leads to the truncation of the C-terminal SET domain. Domains are indicated as follows: AT Hook, DNA-binding AT hooks (green); CXXC, zinc finger containing CXXC motif (yellow); PHD, plant homeodomain fingers (blue/red); F/Y rich, phenylalanine/tyrosine-rich N- and C-terminus (purple/magenta); SET, suppressor of variegation, enhancer of zeste, trithorax domain (orange); and Post SET domain (brown).

*KMT2A* encodes a histone H3 lysine 4 (H3K4) methyltransferase, a critical epigenetic regulator of numerous developmental genes, such as members of the Hox and Wnt families^8,9^. The identified variant truncates the protein before the C-terminal SET domain, which is essential for catalytic methyltransferase activity (Fig. 2c). The premature stop codon is located more than 50 nucleotides upstream of the final exon–exon junction: the resulting transcript is a prime target for nonsense-mediated mRNA decay^3^. This mechanism leads to transcript loss and subsequent protein haploinsufficiency and is the established pathogenesis for most cases of WSS caused by LoF variants^3,5^. The c.10123del variant is classified as pathogenic according to the American College of Medical Genetics and Genomics/Association for Molecular Pathology guidelines, meeting the criteria for PVS1 (null variant in a gene where LoF is a known mechanism), PM2 (absence from population databases) and PP4 (phenotype highly specific for the gene).

Regarding genotype–phenotype correlations, recent studies have begun to elucidate how specific *KMT2A* variants influence clinical outcomes. Lebrun et al. reported that missense variants located in the CXXC DNA-binding domain may lead to a more severe phenotype compared with haploinsufficiency caused by LoF variants, probably due to a dominant-negative effect^10^. By contrast, our patient harbors a frameshift variant leading to a premature stop codon, which is consistent with the classic LoF mechanism and the typical WSS phenotype rather than the severe forms associated with specific missense clusters. Furthermore, Chan et al. expanded the neurodevelopmental spectrum, suggesting that, while LoF and missense variants are both associated with WSS, they share a distinctive profile of autism spectrum disorder characterized by rigid behaviors, regardless of the mutation type^4^. The diversity of WSS is also highlighted by novel findings such as aberrant splicing variants reported by Niu et al., which underscore the complexity of *KMT2A* regulation^11^. Although parental genetic testing was not performed in our case, the patient’s parents are phenotypically healthy with no history of developmental disorders. Given the fully penetrant autosomal dominant nature of Wiedemann–Steiner syndrome, we consider this variant likely de novo, consistent with a sporadic occurrence.

The clinical presentation of our patient aligned well with the multisystemic nature of WSS. Her cardiovascular and extensive gastrointestinal evaluations were unremarkable; however, many individuals with WSS have presented with comorbidities affecting these systems, including congenital heart defects in approximately 33% of cases and constipation in over 60% (refs. ^6,8^). Other frequently reported symptoms included skeletal anomalies such as vertebral fusion (47%), genitourinary anomalies (47%) and ophthalmologic problems such as strabismus (38%)^5,6^. A diagnosis of WSS has implications for management and surveillance. A multidisciplinary approach to managing WSS is essential according to published clinical guidelines^6–8^. For our patient, this involved ongoing nutritional counseling to manage her growth. Growth hormone therapy was considered if severe short stature persisted after nutritional optimization, a treatment that is effective in some patients with WSS^5,8,9^. In addition, early and intensive intervention, including speech therapy, occupational therapy and enrollment in structured educational programs, was initiated to address her global developmental delay. Regular surveillance for potential comorbidities, including ophthalmologic and orthopedic evaluations, is crucial throughout the lives of those with WSS. Although WSS is a well-established clinical entity, the novelty of the present report lies in the documentation and molecular interpretation of a previously unreported *KMT2A* frameshift variant predicted to disrupt the C-terminal SET domain and to cause LoF via nonsense-mediated mRNA decay, consistent with the established haploinsufficiency mechanism.

## hgv Database

The relevant data from this Data Report are hosted at the Human Genome Variation Database at 10.6084/m9.figshare.hgv.3604.
